# Colorectal dysplasia and adenocarcinoma in patients with ulcerative colitis: an experience from a tertiary care hospital

**DOI:** 10.1186/s12957-018-1385-7

**Published:** 2018-04-19

**Authors:** Naila Younus, Mariam Abid, Atif Ali Hashmi, Saher Aijaz, Muhammad Muzzammil Edhi, Ahmareen Khalid Sheikh, Amir Khan

**Affiliations:** 1 Pakistan Institute of Medical Sciences, Islamabad, Pakistan; 2 Liaquat National Hospital and Medical College, Karachi, Pakistan; 3 Shaheed Zulfiqar Ali Institute of Science and Technology, Karachi, Pakistan; 4 Brown University, Providence, RI USA; 5 Kandahar University, Kandahar, Afghanistan

**Keywords:** Ulcerative colitis, Colorectal dysplasia, Colorectal carcinoma

## Abstract

**Background:**

The rationale behind this study was to find out the frequency of dysplasia and colorectal cancer (CRC) in young patients with ulcerative colitis (UC) using histopathological examination. This facilitated early detection of dysplasia and CRC by regular endoscopic biopsies and also guided physicians on appropriate surveillance and management, thus improved outcome.

**Methods:**

It was a prospective cross-sectional study conducted at the Department of Pathology, PIMS, Islamabad. Seventy-six biopsies of already diagnosed cases of UC of young patients aged between 15 and 40 years of either gender were included. Specimens were fixed in 10% buffer formalin, paraffin embedded followed by cutting, slide preparation, and staining with hematoxylin and eosin (H&E) stain, and examined under light microscope. Statistical package for social sciences (SPSS 21) was used for data compilation and analysis. Mean and standard deviation were calculated for quantitative variables. Frequency and percentage were calculated for qualitative variables.

**Results:**

There were 13 (17.2%) patients who were diagnosed with colorectal dysplasia, 3 (4.0%) with indefinite for dysplasia, 8 (10.5%) with low-grade dysplasia, and 2 (2.6%) with high-grade dysplasia. There were three (3.9%) patients who were diagnosed for colorectal carcinoma, one (1.3%) with grade 1, one (1.3%) with grade 2, and one (1.3%) with grade 3 CRC.

**Conclusion:**

Routine biopsies can identify dysplastic epithelium, which is an established sign for synchronized carcinoma with ulcerative colitis, and give the rationale for surveillance of the patients.

## Background

Ulcerative colitis (UC) is a chronic disease leading to inappropriate mucosal immune activation in the large bowel. Histologic defining features of UC include surface ulceration; dense lymphoplasmacytic and neutrophilic infiltrate in the lamina propria, cryptitis, crypt abscesses (collection of neutrophils in the glandular lumen) leading to progressive distortion; and destruction of the glands which show marked decrease in cytoplasmic mucin, goblet cells depletion, and irregular shapes. There are two peak incidences of inflammatory bowel disease; the first peak occurs between 15 and 30 years of age, while the second peak occurs in patients during fifth to eigth decades of life. Questions concerning the course of the disease, its prognosis, and any associated complications are of paramount importance for the patient and the treating physician. The answers to these questions would not only be helpful in guiding treatment, but these findings would also assist the patient in planning their future. The risk for dysplasia and colorectal carcinoma (CRC) is largely influenced by chronicity of the disease, age of diagnosis, past familial record, and evidence of ongoing active colonic inflammation including the area of colonic involvement and concurrent existence of primary sclerosing cholangitis (PSC) [1]. The likelihood of developing dysplasia and CRC increases sixfold in subjects who have inflammatory bowel disease colitis in contrast to others. Histopathologic evaluation is the gold standard for diagnosing both conditions. Dysplasia is graded as follows [2].

**Fig. 1:**
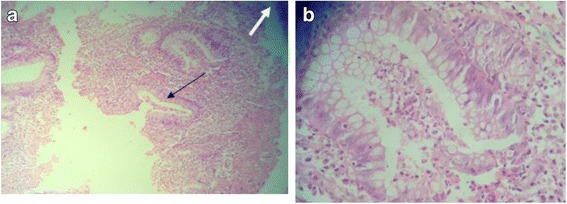
**a** Negative for dysplasia (low power). Ulcerative colitis featuring ulceration, crypt abscesses, lymphoplasmacytic infiltration in the lamina propria (low power). Black arrow: Ulcerative colitis featuring crypt abcess. White arrow: Ulcer **b** Negative for dysplasia (medium power). Ulcerative colitis featuring crypt abscesses (medium power)

**Fig. 2:**
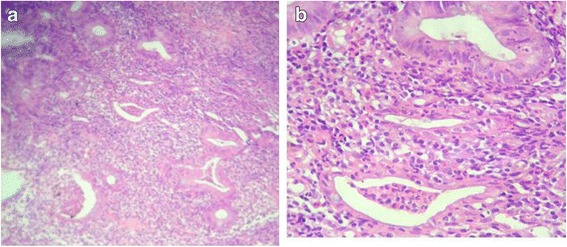
**a** Indefinite for dysplasia (low power). **b** Indefinite for dysplasia (medium power). The epithelium has features suggestive of dysplasia, but changes are insufficient to be unequivocally diagnostic (medium power)

**Fig. 3:**
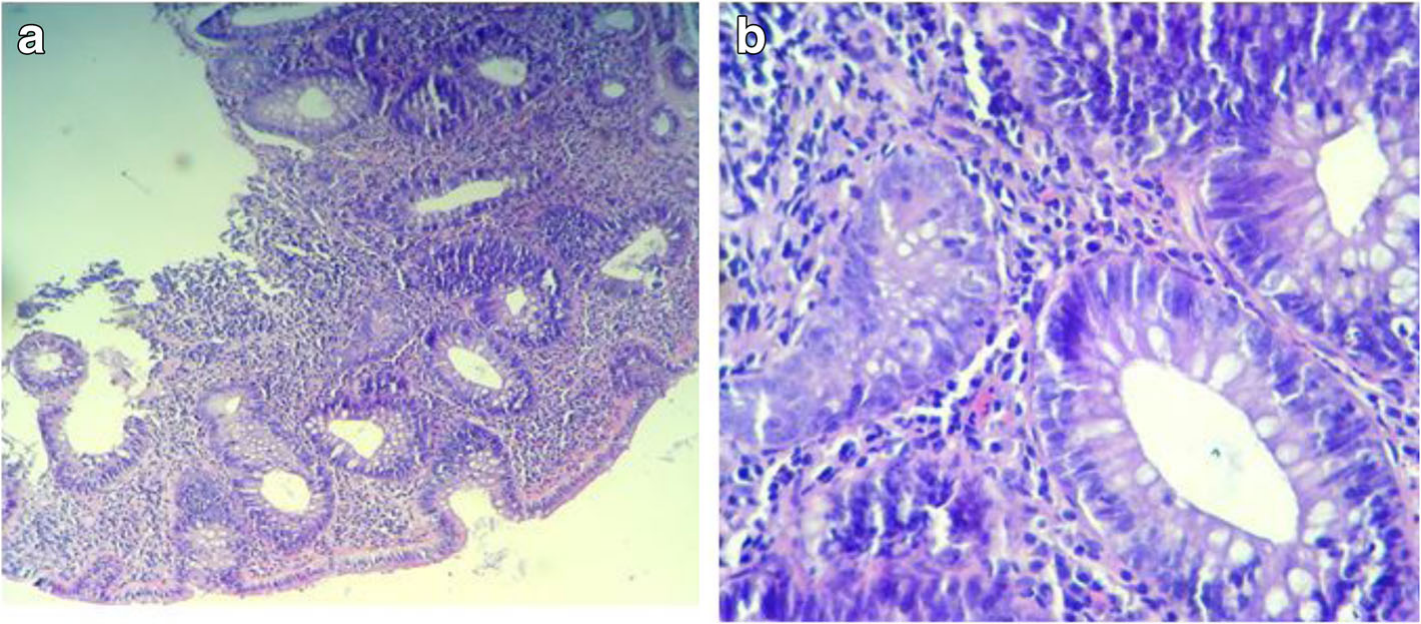
**a** Low-grade dysplasia (low power). **b** Low-grade dysplasia (medium power). Glands lined by cells having hyperchromatic enlarged nuclei with preserved polarity and dystrophic goblet cells (medium power)

**Fig. 4:**
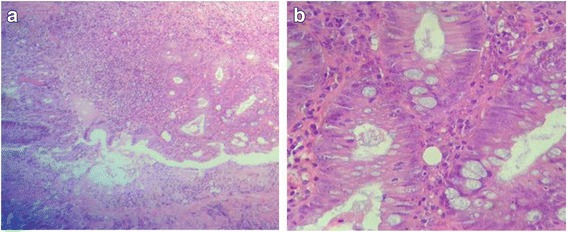
**a** High-grade dysplasia (low power). **b** High-grade dysplasia (medium power). Glands lined by atypical cells having prominent nuclear pleomorphism with hyperchromatic nuclei showing stratifications (medium power)

1. Negative for dysplasia: Inflamed or regenerating mucosa with normal maturation of glandular epithelium. Mitotic figures and histological features of regeneration are confined to the lower half of the glands (Fig. 1)
2. Indefinite for dysplasia: When epithelium has features suggestive of dysplasia but changes are insufficient to be unequivocally diagnostic (Fig. 2)
3. Positive for dysplasia—low-grade dysplasia (LGD): The glands lined by cells having hyperchromatic, enlarged nuclei with preserved polarity, mucinous differentiation is decreased, and dystrophic goblet cells. Atypia may focally reach the surface (Fig. 3)
4. Positive for dysplasia— high-grade dysplasia (HGD): The glands lined by atypical cells having prominent nuclear pleomorphism with hyperchromatic often rounded nuclei that are stratified throughout the cells. The glands showing branching architecture. Atypia extends to the surface (Fig. 4)

**Fig. 5:**
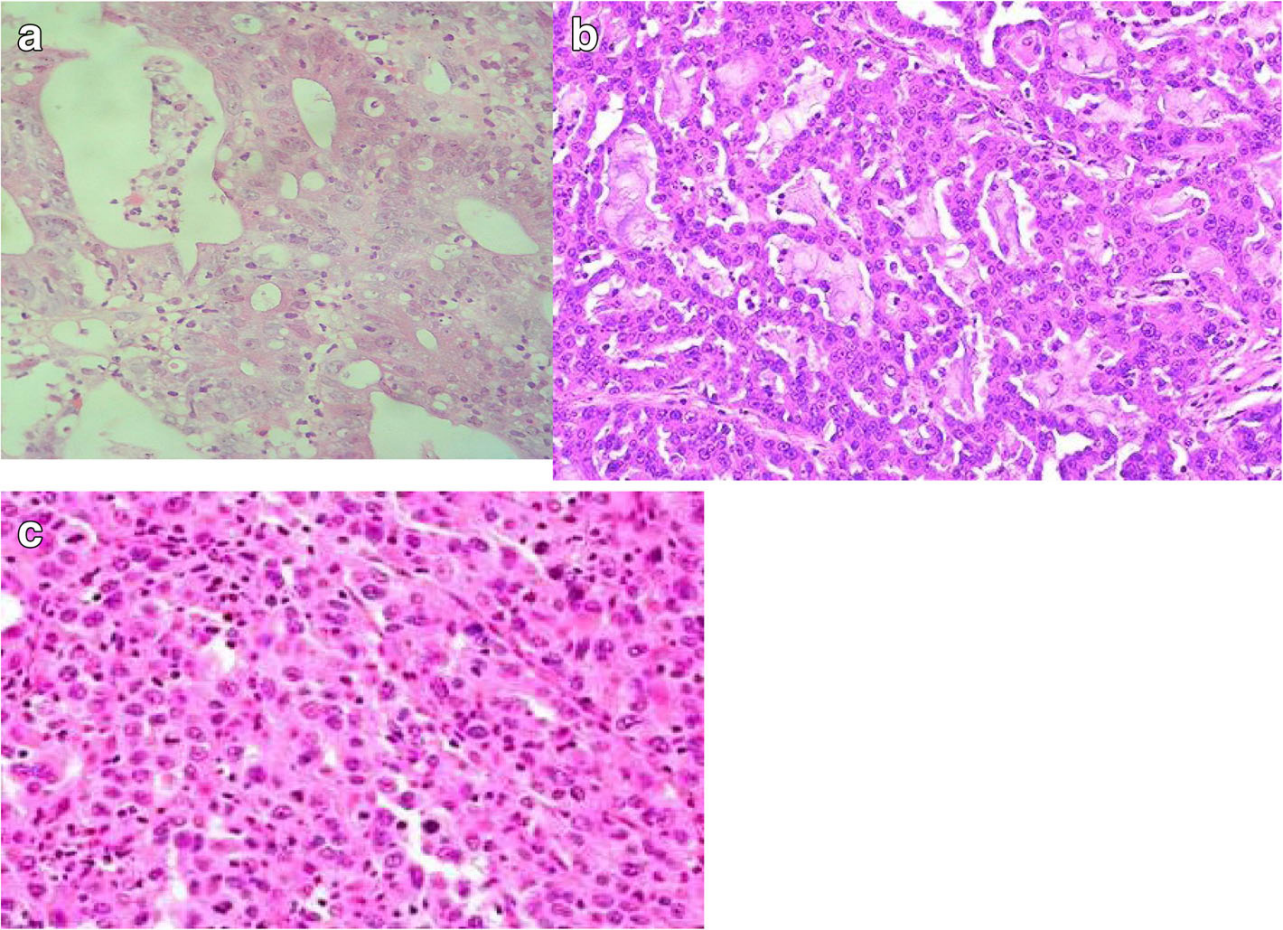
**a** CRC grade 1. Glands lined by anaplastic cells in the desmoplastic stroma (medium power). **b** CRC grade 2. Less well-formed glands with focal cribriform architecture (medium power). **c** CRC grade 3. Tumor grows on solid sheets with no distinct gland formation (medium power)

Also, UC predisposes to a higher incidence of several other synchronous colorectal cancers. UC-induced CRC occurs in a younger age group compared to sporadic CRC. Moreover, young patients who are diagnosed with pancolitis have a 30% absolute risk of developing CRC after 35 years of diagnosis [2]. CRC is a fatal long-lasting outcome of chronic UC and is the result of a complex series of molecular and histological abnormalities of the intestinal lining characterized by a gland of variable differentiation and lined by anaplastic cells having large, hyperchromatic nuclei and prominent nucleoli with prominent mitotic activity often with atypical forms. CRC is graded as follows:

- Grade 1: Composed predominantly of well-formed glands lined by anaplastic cells, in a desmoplastic stroma (Fig. 5a).
- Grade 2: Less well-formed glands with focal cribriform architecture (Fig. 5b).
- Grade 3: Tumor grows in solid sheets with no distinct gland formation (Fig. 5c).

Ulcerative colitis is a type of inflammatory bowel disease in which there are relapsing and remitting episodes of inflammation confined to the mucosa of the colon. It almost always involves the rectal area and may spread proximally and in a continuous manner to occupy other parts of the colon. The Mayo scoring system can also be used to assess disease severity and monitor patients during therapy [3]. Scores vary from 0 to 12 and higher score demonstrating increased severity of the disease. Evaluation of a patient with suspected ulcerative colitis serves to exclude other causes of colitis, establish the identification of UC, and to determine the degree of involvement and grades of dysplasia. The likelihood and timing of colectomy rely on the area involved and grades of dysplasia or carcinoma at presentation [4, 5]. Individuals with the disease have greater chances to develop colonic carcinoma. The risk of carcinoma appears to be highest in patients with pancolitis, while those with the involvement of rectum and rectosigmoid areas are probably not at greater risk of CRC, regardless of the duration of disease [6]. The CRC risk starts to rise 8 to 10 years after the commencement of disease in patients with pancolitis [7, 8].

Other factors that are related to an increased risk of cancer include endoscopic and histological degree of inflammatory reactions, presence of sporadic colorectal cancer in first degree relatives (doubles the risk), postinflammatory pseudopolyps (twofold increased risk), and the presence of primary sclerosing cholangitis (fourfold increased risk) [9, 10].

Dysplasia in UC is preceded by a long-standing chronic inflammatory reaction and can be established at distant sites from cancer. In contrast, dysplasia in sporadic colon cancer is usually associated with a discrete polyp without inflammation. The purpose of the majority of surveillance plans has been the recognition of dysplasia, which is related to greater chances of developing carcinoma [11, 12]. There are also facts that in patients who undergo surveillance, cancers are likely to be diagnosed at the initial stage, and likewise, these patients have a better outcome. Shivakumar et al. [13] aimed to assess the outcome of a newly initiated pilot screening program for screening CRC among UC patients in India. In their prospective study from an academic hospital setting, patients with UC at high risk of CRC were offered screening by magnifying chromocolonoscopy, and the frequency of neoplastic lesions was assessed. They found that on initial screening, low-grade dysplasia (LGD) was seen in five (17.2%) and high-grade dysplasia (HGD) in three (10.3%). Of these three, one accepted proctocolectomy immediately, one underwent surgery for adenocarcinoma, and one refused surgery. Twelve follow-up colonoscopies in nine patients revealed three new LGD. They concluded that high-grade dysplasia and subsequent adenocarcinoma can be detected with careful follow-up in Indian patients with long-standing UC, but acceptance of surveillance and subsequent therapy are suboptimal.

The possibility of having CRC in subjects with ulcerative colitis depends upon the duration of illness which is 2% at 10 years, 8% at 20 years, and 18% at 30 years [14]. The first report of intestinal cancer occurrence in IBD was published over 80 years ago. Since then, numerous studies have addressed this issue, but the true risk of malignancy remains uncertain. Current screening endoscopy protocols are primarily based on white light endoscopy (WLE) and random biopsies. Novel endoscopic techniques include chromoendoscopy (CE) and confocal laser endomicroscopy (CLE) [15]. On account of these screening techniques, sufficient evidence is available in the literature today suggesting a greater risk of colonic cancer in subjects having long-standing ulcerative colitis [15, 16].

According to available data, patients who undergo cancer surveillance are more likely to be diagnosed at the initial stage, and therefore, these patients exhibit better outcomes as compared to patients diagnosed at an advanced stage. Also, improved clinical outcomes are reported in 27% of cases of CRC and HGD, who had colectomy within 6 months of early detection of flat low-grade dysplasia [16, 17]. There is indirect support that surveillance is possibly useful and cost-effective method to decrease mortality from colonic carcinoma associated with ulcerative colitis [14–16]. The rationale behind this study was to establish the frequency of dysplasia and colorectal cancer in young (15–40 years) patients with UC on histopathological examination. This study facilitated the early detection of CRC by regular endoscopic biopsies which guided the physicians on appropriate management, thus improved patient’s outcome. The objective of this study was to determine the frequency of colorectal dysplasia and colorectal cancer in young (15–40 years) patients with ulcerative colitis using histopathological examination techniques.

## Methods

It was a prospective cross-sectional study conducted in the Histopathology Unit, Department of Pathology, Pakistan Institute of Medical Sciences (PIMS), Islamabad, including 76 patients of ulcerative colitis which underwent biopsies from January 2016 to June 2017 over a period of 1 year and 6 months. Sample size was 76 by consecutive sampling (non-probability) (calculated by using WHO sample size calculator taking a level of significance = 95%, anticipated population proportion = 27%, absolute precision required = 10%, sample size *n* = 76 patients). All young patients (15–40 years old) with confirmed UC diagnosis were included. The patients were selected irrespective of their gender. Exclusion criteria comprised of previously diagnosed cases of colorectal malignancy, patients on chemotherapy, and any contraindication to biopsy.

Biopsies of patients were collected from the outdoor department of pathology, Pakistan Institute of Medical Sciences (PIMS), Islamabad. Patient’s demographic data along with registration number, presenting complaints, and clinical profile were recorded. The study was approved by the research and ethical review committee of Liaquat National Hospital, and informed written consent was taken from all patients at the time of surgery. After the informed consent, patients were included in the study on the basis of clinical signs and symptoms of ulcerative colitis. All the biopsy specimens were immediately fixed in 10% buffer formalin. Following gross examination, the tissue was paraffin embedded, followed by cutting, slide preparation, and staining with hematoxylin and eosin (H&E) stain. Macroscopic and microscopic findings were commented upon. Slides for histopathology were examined under light microscopy by consultant pathologist and postgraduate medical student independently, and the diagnosis was recorded.

Statistical package for social sciences (SPSS 21) was used for data compilation and analysis. Mean and standard deviation were calculated for quantitative variables. Frequency and percentage were calculated for qualitative variables.

## Results

### Demographic profile of the respondents

Seventy-six (76) biopsies of diagnosed cases of UC of young patients (15–40 years) of either gender were included in the study. Fifty patients (65.8%) were males with the mean age of 28.88 years ± 7.22 SD, and 26 (34.2%) were females with the mean age of 26.38 years ± 7.26 SD. Cumulative mean age was 28.03 years ± 7.33 SD.

### Frequency of dysplasia and CRC in the study population

From recruited 76 patients, 60 (79%) patients were negative for any dysplasia or colorectal carcinoma as per our operational definition. There were 13 (17.10%) patients who were diagnosed with dysplasia as per our operational definition. Out of 76 patients, 3 (3.94%) presented with indefinite for dysplasia, 8 (10.53%) with low-grade dysplasia, and 2 (2.63%) with high-grade dysplasia. There were three (3.94%) patients who were diagnosed with colorectal carcinoma as per our operational definition, one (1.32%) with grade 1 CRC, one (1.32%) with grade 2 CRC, and one (1.32%) with grade 3 CRC. Results are shown in Table 1.

**Table 1:** Frequency of dysplasia and colorectal carcinoma in patients with ulcerative colitis (sample size 76)

Diagnosis	Frequency	Percentage (%)
Negative for dysplasia	60	78.95
Indefinite for dysplasia	3	3.94
Low-grade dysplasia	8	10.53
High-grade dysplasia	2	2.63
CRC grade 1	1	1.32
CRC grade 2	1	1.32
CRC grade 3	1	1.32

## Discussion

In the present study, we aimed to evaluate the frequency of malignant and premalignant lesions in patients presenting with UC and found 17 and 3.9% frequencies of dysplasia and CRC, respectively, indicating the need for regular endoscopic follow-up with histological evaluation in patients with UC in locoregional population.

Rubin et al. [18] have recognized that dysplasia and colonic cancer in ulcerative colitis occurred by means of ways different from sporadic cancer and may arise in flat mucosa similar to the adjacent area. They also established the surveillance recommendations and stressed the approach of regular colonoscopic evaluations and regular random biopsies of susceptible areas [18]. They observed that there were 1339 surveillance assessments in 622 cases of ulcerative colitis. Forty-six patients had dysplasia or colonic cancer at a mean age of 48 years and with a mean length of illness of 20 years. These individuals went through 128 surveillance assessments (mean 3/patient), and in 51 assessments, 75 cases of separate dysplasia or carcinoma were detected. Thirty-eight out of 65 cases of dysplasia (58.5%) and 8 out of 10 cases of colonic carcinoma (80.0%) were visible endoscopically as 23 polyps and masses, 1 stricture, and 22 abnormal mucosae. Blonski et al. [19] intended to find out whether dysplasia is detectable through regular colonoscopic surveillance by assessing only those who had dysplasia without obvious colonic cancer [19]. They systematically reviewed their medical records and endoscopic pathologic records between 1997 and 2004 at the University of Pennsylvania Health System. They found that of the 113 individuals with colonic dysplasia established by histopathology at their hospital, 102 (90%) had ulcerative colitis. On the whole, 58 foci of dysplasia were identified; 51 (88%) were seen endoscopically, and 7 (12%) were endoscopically indiscernible. They found that the majority of the dysplastic lesions in ulcerative colitis were colonoscopically identified, but more prospective research of more patients is required to confirm the recent results. Their observations have the potential to amend recent proposals for surveillance biopsy specimens in ulcerative colitis if confirmed by prospective researches. Rutter et al. [20] reviewed the proportion of dysplastic lesions that have been identified colonoscopically in patients who went through colonoscopic surveillance at their institute [20]. They performed a retrospective analysis of endoscopically identified dysplastic lesions or carcinoma in individuals with UC who went through surveillance from 1988 to 2002. Well-known surveillance recommendations were applied in all patients that incorporated non-targeted segmental biopsies every 10 cm all through the colonic length, besides targeted biopsy samples of colonoscopically visible areas. Identification of cancer foci was carried out through non-random as well as random biopsy specimen. They observed that an overall 525 patients went through 2204 colonoscopic surveillance. One hundred ten carcinomatous foci were identified in 56 cases: 85 (77.3%) were macroscopically evident at endoscopy, and 25 (22.7%) were not evident on colonoscopic evaluation.

In our study, LGD was found in eight (10.5%) and HGD and CRC in five (6.5%). This is in accordance with the observations made by Navaneethan et al. [21] that aimed to study the risk of progression of LGD to advanced neoplasia (AN), referred to as HGD or CRC for UC subjects undergoing surveillance based on location and morphology of LGD [21]. They studied 997 UC patients who underwent 3152 surveillance colonoscopies from 1998 to 2011 and calculated Kaplan-Meier estimates and incidence rates. They found that of the 102 (10.2%) patients with LGD (65 raised and 37 flat), 5 (4.9%) patients progressed to AN (3 HGD and 2 CRC) following a median follow-up of 36 months (interquartile range 18–71 months).

In our study, the mean age of patients was 28.03 years ± 7.26 SD, this is quite comparable with the study done by Pekow et al. [22] who evaluated patients with the mean age of 27.0 years ± 12.5 SD from a sole organizational referral center who had a history of UC and a diagnosed either as low-grade dysplasia or indefinite dysplasia from 1994 to 2008 as established by two skilled gastrointestinal histopathologists [22]. They included 35 subjects in the study, of whom 2 subjects with indefinite dysplasia and 2 with low-grade dysplasia progressed to high-grade dysplasia or colorectal carcinoma with a mean time period of 49.8 months and observed that overall, the incidence rate for AN for every subject was 2.7 cases of high-grade dysplasia or colorectal carcinoma for every 100 individuals-years at risk. For fLGD and polypoid low-grade dysplasia, the incidence rate of AN was 4.3 and 1.5 cases for every 100 individuals-years at risk, respectively.

One of the limitations of the study was the duration of disease, and long-term follow-up was not known. Therefore, we suggest a more detailed study evaluating the duration of affecting development of dysplasia and CRC.

## Conclusion

We aimed to evaluate the frequency of dysplasia and CRC in patients with UC in locoregional population and found 17 and 3.9% frequencies of dysplasia and CRC, respectively, in the present study. Regular colonoscopic evaluations and biopsies can identify epithelial dysplasia, a recognized indication for a synchronized malignant lesion in patients with ulcerative colitis, and provide the rationale for surveillance of these subjects. Colorectal carcinoma can also be identified at an initial phase, and the outcome is proper management of these patients.
